# Probable enterotoxin-associated toxic shock syndrome caused by *Staphylococcus epidermidis*

**DOI:** 10.1186/s12887-023-03914-5

**Published:** 2023-03-07

**Authors:** William F. Pomputius, Samuel H. Kilgore, Patrick M. Schlievert

**Affiliations:** 1 Division of Infectious Disease, Children’s Minnesota, Minneapolis, MN 55455 USA; 2 Department of Microbiology and Immunology, Carver College of Medicine, University of Iowa, 51 Newton Road, Iowa City, Iowa, 52242 USA; 3 Department of Internal Medicine, University of Iowa Carver College of Medicine, Iowa City, IA 52242 USA

**Keywords:** Case report, *Staphylococcus epidermidis*, Enterotoxin, Toxic shock syndrome, Urinary tract infection

## Abstract

**Background:**

We describe a case of a toxic shock-like syndrome in a child, which was associated with *Staphylococcus epidermidis* instead of *Staphylococcus aureus* or *Streptococcus pyogenes*, the usual causes of toxic shock syndrome.

**Case presentation:**

The patient was an 8-year-old boy who developed a toxic shock syndrome-like illness, including fever, hypotension, and rash. The *Staphylococcus epidermidis* isolate was cultured from urine, but this organism was unavailable for toxin testing. Multiple blood cultures were negative. Instead, a highly novel assay was used on acute plasma from the patient which demonstrated the presence of the genes for superantigens, staphylococcal enterotoxins A, C, D, and E. Superantigens are the known causes of toxic shock syndrome.

**Conclusions:**

Our study suggests strongly that *Staphylococcus epidermidis* was causing the TSS symptoms through the known *Staphylococcus aureus* superantigens. It is unknown how many other such patients exist; this should be explored. Of great importance is that PCR performed directly on blood plasma in the absence of microbial isolation could be used to demonstrate superantigen genes.

## Background

Toxic shock syndrome (TSS) is a serious infection caused by *Staphylococcus aureus* [1, 2] and *Streptococcus pyogenes* [3–5], with occasional cases associated with Groups B, C and G streptococci [6–9]. Superantigens, which include toxic shock syndrome toxin-1 (TSST-1), staphylococcal enterotoxin serotypes B and C, and streptococcal pyrogenic exotoxin serotypes A and C, are the major causes of TSS [7, 8].

All menstrual TSS is caused by TSST-1 [10], most likely because of its ability to easily penetrate mucosal surfaces [11]. Staphylococcal enterotoxins B and C and streptococcal pyrogenic exotoxins A and C, as well as TSST-1, are associated with non-menstrual TSS [7, 8, 12, 13].

Many coagulase-negative staphylococci have been examined for production of TSST-1 [14, 15]. However, none so far has been found to be positive for either the protein or the *tstH* gene that encodes the toxin. However, occasionally coagulase-negative staphylococci produce enterotoxins. The FRI909 organism, used by many researchers studying staphylococcal enterotoxins, to produce large quantities of staphylococcal enterotoxin C, is a coagulase-negative staphylococcus [16]. The production of staphylococcal enterotoxins by coagulase-negative staphylococci raises the possibility that non-menstrual TSS cases may be associated with such organisms. This study presents a case in a male patient consistent with coagulase-negative staphylococcal TSS.

## Case presentation

An 8-year-old boy was admitted for a 1-day history of headache, vomiting, extreme fatigue and fever, initially 102 °F, along with photophobia and hyperacusis. He complained once of sore throat, but had no abdominal pain, diarrhea, or urinary tract symptoms. Shortly before admission, his fever rose to 106.4 °F, accompanied by confusion and hallucinations. Mild conjunctival injection and a petechial rash on the cheeks were seen on admission but resolved in less than 24 hours. He was briefly hypotensive with diastolic blood pressures as low as 37 mmHg; his hypotension responded to normal saline fluid boluses alone.

Initial laboratory studies (Table 1) were significant for white blood cell count of 21.0 (differential of 92% neutrophils, 1% bands, 2% lymphocytes, and 5% monocytes). Platelet count was 164,000. Procalcitonin was elevated at 21.52 ng/mL, while C-reactive protein was elevated at 19.59 mg/dL. A creatinine level of 0.5 mg/dL was elevated for his weight, with his baseline later determined to be 0.34–0.37 mg/dL. Hepatic function testing was normal with the exception of alkaline phosphatase (Table 1). Creatine phosphokinase was mildly elevated at 297 U/L. His albumin was normal upon admission (4.1 g/dL) but dropped (to 2.3 g/dL) by day 4 of hospitalization. Coagulopathy was present on admission: prothrombin time (PT) was 16.5 second with fibrinogen mildly elevated at 476 mg/dL. Elevated levels of interleukin-1β, interleukin-6, interleukin-8, and tumor necrosis factor-α were present in blood.

**Table 1:** Patient laboratory values on admission or as indicated

Laboratory Test	Patient Value^a^	Normal Range
White Blood Cell Count	**21 × 10**^**3**^**/mm**^**3**^	4.5–11.0 × 10^3^/mm^3^
Neutrophils	**92%**	45–75%
Bands	1%	
Lymphocytes	**2%**	16–46%
Monocytes	5%	4–11%
Platelets	**164 × 10**^**3**^**/μl; decreased to 100 × 10**^**3**^**/**μl **by day 2 of hospitalization**	130–400 × 10^3^/μl
Procalcitonin	**21.52 ng/ml**	0–0.09 ng/ml
C-reactive protein	**19.59 mg/dL**	0–0.5 mg/dL
Creatinine	**0.5 mg/dL**	Baseline: 0.34–0.37 mg/dL
Creatine phosphokinase	**297 U/L**	21–232 U/L
Total Bilirubin	0.4 mg/dL	0–1.0 mg/dL
Alkaline Phosphatase	**207**	45–115 U/L
AST (Aspartate Aminotransferase)	30 U/L	0–40 U/L
ALT (Alanine Aminotransferase	20 U/L	10–55 U/L
Albumin	**4.1 g/dL; decreased to 2.3 g/dL by day 4 of hospitalization**	3.1–4.3 g/dL
Prothrombin Time (PT)	**16.5 seconds**	8.5–12.4 seconds
Fibrinogen	**476 mg/dL**	200–400 mg/dL

^a^Bold indicates patient values different from normal values

Admission blood culture (and multiple additional blood cultures) were negative, as was a nasal culture for *S. aureus* and *Streptococcus pyogenes*. Nasal cultures were done because the patient was orally intubated at the time of the infectious disease consultation, and throat swab for PCR or culture was not possible. There is precedent for using nasal cultures, as well as pharyngeal cultures, for detection of the Group A streptococcal carrier state [17]. Nasal swab for methicillin-resistant *S. aureus* by PCR was negative, as was a throat swab for *Streptococcus pyogenes* by PCR. Urine culture (voided from a circumcised male) grew greater than 100,000 colonies/mL of *S. epidermidis*, resistant to oxacillin and penicillin, and sensitive to trimethoprim-sulfamethoxazole, gentamicin, and nitrofurantoin; urinalysis showed a specific gravity of 1.020, pH of 3.5, and dipstick positive for albumin and small ketones. Microscopic analysis showed 0–5 white blood cells and 0–2 red blood cells per high power field. After intubation for sedation and procedures, endotracheal culture showed growth of only normal upper respiratory flora (Viridans group streptococci, gamma-hemolytic streptococci, and *Stomatococcus*). A respiratory pathogen PCR panel (BioFire, St. Lake City, UT) was negative for adenovirus, *Bordetella parapertussis* and *Bordetella pertussis*, *Chlamydia pneumoniae*, coronaviruses 229E, HKU1, NL63, OC43, SARS-CoV-2, human metapneumovirus, influenza A and B, *Mycoplasma pneumoniae*, parainfluenza 1–4, and respiratory syncytial virus and positive for rhinovirus/enterovirus, which did not appear to be clinically relevant. SARS-CoV-2 antibody test was negative. Cell-free DNA metagenomic assay of plasma (Karius, Redwood City, CA) was negative.

Lumbar puncture obtained clear cerebrospinal fluid, with the following indices: 0 white blood cells, 2 red blood cells, glucose 78, and protein 15. Gram stain and bacterial culture were negative; a meningitis/encephalitis PCR panel (Quest, Minneapolis, MN) was negative. Additionally, serum herpes simplex virus DNA PCR was negative. Serum varicella zoster virus DNA PCR was not done.

The patient’s voided urine culture was initially considered a contaminant (despite its pure growth at high colony count), so treatment was directed against possible viral and bacterial causes of encephalopathy. This included vancomycin (hospital day 1–5), ceftriaxone (hospital day 1–8), clindamycin (hospital day 1–5), and acyclovir (hospital day 2–4). Doxycycline was begun on hospital day 4 and continued for a 10-day course, given concern for possible *Chlamydia* or *Mycoplasma* encephalitis. In retrospect, doxycycline might well have been active against coagulase-negative staphylococci.

On the empirical therapy (vancomycin, ceftriaxone, clindamycin, and acyclovir), the patient became afebrile within 72 hours. White blood cell count had normalized to 7.1 by hospitalization day 2 (Table 1); at that time his platelet count reached its nadir (Table 1), and subsequently returned to normal. No intravenous immunoglobulin or steroids were given. Plasma was obtained from residual of other ordered tests for later study.

Abdominal ultrasound on hospitalization day 3 showed mild right collecting system dilatation and an unremarkable bladder. Besides the complete abdominal ultrasound, a negative chest X-ray was obtained on hospital day 1. Repeat chest X-ray (portable) showed perihilar and left basilar opacities, read as a combination of atelectasis and edema. Confusion and hallucination persisted after fever resolution and removal of sedation. MRI of the brain, including MRA and MRV, was negative.

Further neurological workup included negative cerebrospinal fluid autoantibodies (AChR ganglionic neuronal, AMPA-R CBA, Amphiphysin, Antiglial nuclear [AGNA-1], Antineuronal nuclear [ANNA-1], Antineuronal nuclear [ANNA-2], Antineuronal nuclear [ANNA-3], CASPR2 IgG CBA, CRMP-5 IgG, DPPX Ab IFA, GABA-B-R Ab IFA, GAD65, GFAP IFA, IgLONS IFA, LGI1 IgG CBA, mGlur1 Ab IFA, NIF IFA Ab, NMDA-R Ab CBA, N-type Calcium channel, P/Q Type Calcium channel, Purkinje Cell Cytoplasma Ab T1 [PCA-1], Purkinje Cell Cytoplasm Ab T2 [PCA-2], Purkinje Cell Cytoplasma Ab TTr [PCA-Tr], and Smooth Muscle) as performed by the Mayo Clinic Laboratory. Additional autoimmune antibodies tested, not on the panel above, included: Proteinase 3 AB, Myeloperoxidase Ab, DNA double stranded Ab, and Rheumatoid factor. All were negative.

Since the patient presented with sepsis and encephalopathy in Minnesota in October, tick-borne pathogens and arboviruses were considered. Arbovirus antibodies were assayed in both serum and cerebrospinal fluid with negative results. Our arbovirus panel contains the following viruses: Eastern Equine, Lacrosse/California, St. Louis, and Western Equine. In addition, cerebrospinal fluid assays for West Nile Virus IgG and IgM were negative. Tick-borne pathogen antibody panel and tick-borne pathogen PCR panel (Mayo Clinic Laboratory), and *Bartonella* and *Chlamydophila* antibodies were negative. Mycoplasma serologies were repeated 3 weeks after the inpatient results were obtained. Mycoplasma IgG by IFA was negative both acutely and in convalescence; IgM was positive acutely and remained positive in convalescence. These results demonstrated that the initial IgM results were false-positive or cross-reactive.

Propofol and midazolam were used for sedation. No other CNS medications were used, as the boy’s sensorium gradually cleared after 7 days of hospitalization. He completed a 10-day course of ceftriaxone. Doxycycline was also prescribed for 10 days to cover possible infection with either atypical bacteria or *Staphylococcus species* and completed as an outpatient. Seen in clinic 4 weeks after discharge, he was fully recovered. Besides *Mycoplasma* serologies, SARS-CoV-2 and Lyme antibodies were also repeated and remained negative.

**Fig. 1:**
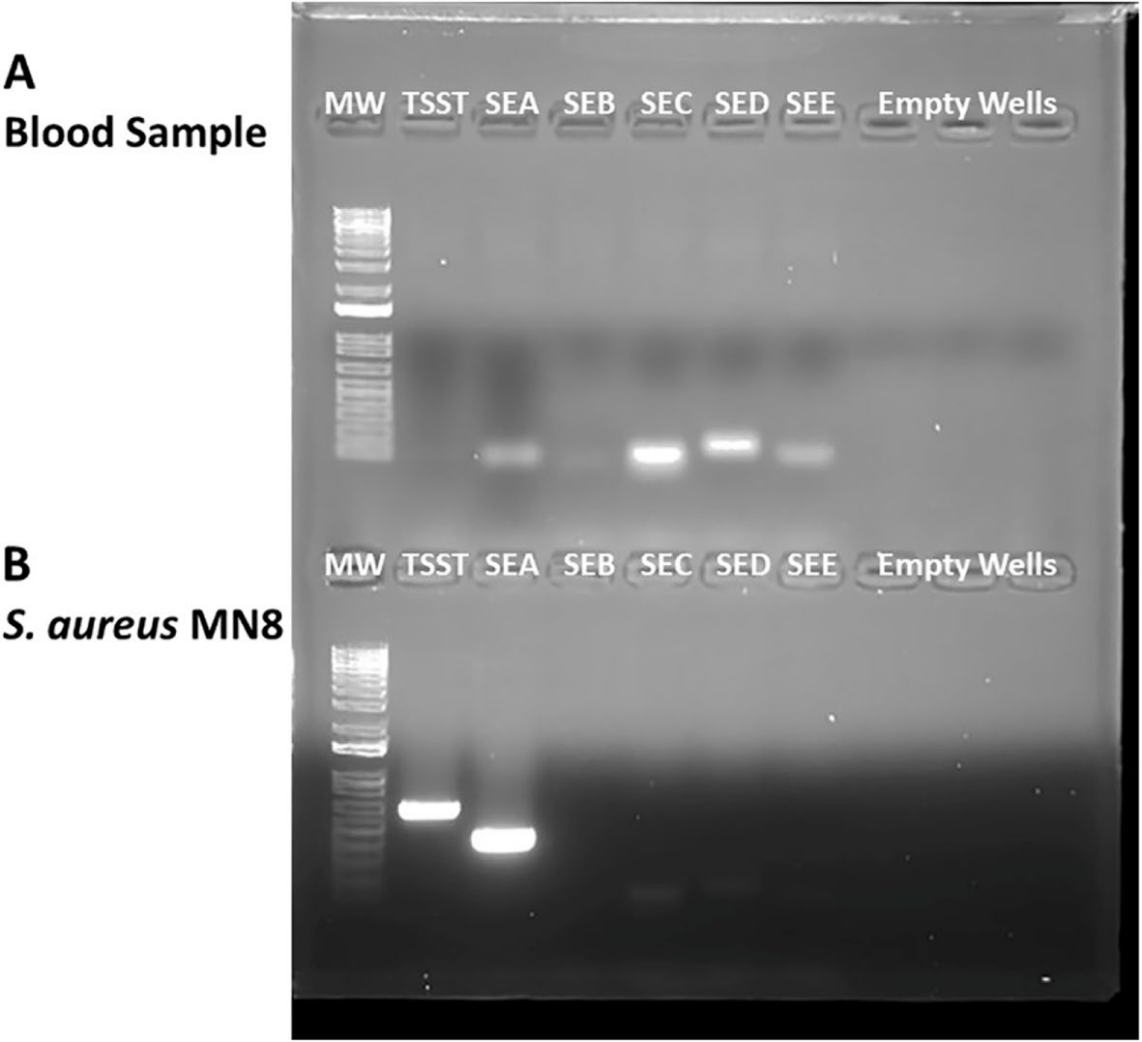
PCR on (**A**) patient blood plasma and (**B**) *S. aureus* strain MN8 DNA for detection of staphylococcal superantigen genes encoding TSST-1 and staphylococcal enterotoxins (SEs) A, B, C, D, and E. Bands indicate the presence of PCR products for the indicated superantigen. Strain MN8 is known to have the genes for TSST-1 and SEA, but not SEB-SEE. MW = molecular weight standards. The gel shown has not been modified from its original appearance as photographed

Residual plasma was recovered and sent to author Patrick M. Schlievert where PCR analyses were performed specifically for the superantigens, staphylococcal enterotoxins A-E [18]. The positive control was DNA from *S. aureus* MN8, a menstrual TSS isolate. The blood plasma produced positive PCR results for staphylococcal enterotoxins A, C, D, and E (Fig. 1A). There was a very faint band for staphylococcal enterotoxin B which likely results from staphylococcal enterotoxins B and C being 75% identical; thus there may be minor cross-reactivity [19]. There was no PCR product for the TSST-1 gene, suggesting the positive PCR results for other superantigens were not the result of nonspecific PCR reactions. The MN8 strain produces the superantigens TSST-1 and staphylococcal enterotoxin A; the strain is negative for staphylococcal enterotoxins B, C, D, and E (Fig. 1B). Additionally, we have extensively performed PCR on urine and other body fluids from non-TSS individuals with no PCR products observed.

## Discussion and conclusions

*Staphylococcus epidermidis* and other coagulase-negative staphylococcal species are most often recognized as pathogens, either in prematurely born infants or in settings of indwelling medical devices, including central lines or bladder catheters [20]. In the absence of such risk factors, certain coagulase-negative staphylococci such as *S. saprophyticus* are well recognized to cause urinary tract infections in young women [21]. *S. epidermidis* urinary tract infections in older adults, by contrast, are related to bladder catheterization and can be complicated by bacteremia [22]. Pediatric case reports suggest that *S. epidermidis* can cause pyelonephritis in children with no predisposing risk factors, and caution is warranted that this organism not be uniformly dismissed as a skin contaminant [23–25].

In our patient, with negative blood cultures, the question remains as to the significance of the pure urine culture of *S. epidermidis*. Even if discounted as a cause of bacteriuria, its presence as a skin colonizer may hold clinical significance in this child, whose clinical presentation including persistent hallucinations is consistent with a toxigenic illness (toxic shock syndrome-like) [26], in whom no other convincing pathogen could be determined, and whose timing of clinical recovery was consistent with an expected response to antibiotics. Neurologic abnormalities are common in cases of TSS [27]. It is formally possible, but highly unlikely, that there was some obscure locus of *S. aureus* infection which was not observed.

Coagulase-negative staphylococci may be under-appreciated as a cause of toxic shock syndrome. It has been shown many times that such coagulase-negative staphylococci do not produce TSST-1 [14, 15]. However, coagulase-negative staphylococci are capable of secreting a number of staphylococcal enterotoxins and cytotoxins, normally produced by *S. aureus* [16, 20, 25].

The isolation of staphylococcal toxins from infant urine samples has been postulated as a possible marker of transient bacteremia [28]; toxin genes have been detected by multiplex PCR in *S. aureus*, both methicillin-sensitive and methicillin-resistant, causing urinary tract infections on a urology ward [29]. In our patient, the detection in plasma of staphylococcal superantigen genes in the presence of a urinary tract coagulase-negative staphylococcus, with potential for producing such toxins, may point to a similar process. It is important that the genes for staphylococcal enterotoxins A, C, D, and E were detected in the plasma of the patient. Staphylococcal enterotoxins A, D, and E are highly related to each other, and the results possibly reflect cross-reactivity [7]. These three superantigens are typically produced in low concentrations, usually in the nanogram to picogram per milliliter range [7]. As little at 0.1 μg of staphylococcal superantigens can cause TSS symptoms [30].

On the other hand, the three major superantigens associated with staphylococcal TSS are TSST-1 and staphylococcal enterotoxins B and C; the gene for staphylococcal enterotoxin C was detected in the plasma of the patient in this report [7]. These latter three superantigens can be produced in concentrations up to 20,000 μg/ml in thin-films as might be expected on many human body sites [31].

Many studies have addressed the mechanism by which superantigens cause TSS. All superantigens have the overall shape of a kidney bean of 35 × 50 angstroms [7]. Superantigens are typically viewed in standard structural displays with the kidney bean shape laying on its side with the two arms pointing up. All superantigens have the ability to bind to major histocompatibility complex II (MHC II) molecules with low affinity on the right side of the kidney bean, while some superantigens have this low affinity right side site but also have a high affinity MHC II site on the left side [7]. TSST-1 and staphylococcal enterotoxins B and C bind only through the low affinity MHC II site on antigen-presenting cells, that is then cross-bridged with T lymphocyte receptors of up to 50% of T cells [7, 8]. The T lymphocyte receptor site on superantigens is in the groove at the top of the kidney bean shape. These same three superantigens are produced in high amounts compared to other superantigens, as high as 15 mg/ml in biofilms, as might be present on human mucosal surfaces [31]. The consequence of the interaction of these three superantigens with MHC II and T lymphocyte receptors is massive cytokine production, including interleukin-1β and tumor necrosis factors [7, 8, 32]. These two cytokines then induce what is referred to as the “original” cytokine storm in humans, including fever (interleukin-1β) and hypotension (tumor necrosis factors-α and β), the major clinical features of TSS and as observed in the patient in this report [33]. TSST-1 appears to interact strongly with another immune co-stimulatory receptor on epithelial cells (CD40) that explains its unique association with menstrual TSS [11].

Staphylococcal enterotoxins A, D, and E, like all superantigens, interact with the low affinity MHC II receptor on antigen-presenting cells, but these three superantigens have the additional high-affinity MHC II receptor on antigen-presenting cells (left side of the kidney bean shape) [7, 8]. All three also interact with T lymphocyte receptors. The presence of the high affinity MHC II site increases the activity of these superantigens by 10-fold compared to TSST-1 and enterotoxins B and C, but likely not enough to offset their being expressed by staphylococci in only low amounts (< 1 μg/ml) [8]. These latter three superantigens may thus be more important as colonization factors than agents produced in high enough concentration to cause TSS [34].

Of overall key importance to our study is that neither *S. aureus* nor *Streptococcus pyogenes*, the two classic causes of TSS, was isolated from the patient. The patient in this study had an infection with *S. epidermidis.* Although superantigen proteins were not isolated from the bloodstream, four superantigen genes were detected in the plasma. This suggests strongly that the coagulase-negative staphylococcus found in the urine was causing the TSS symptoms through the known *S. aureus* superantigens. It is unknown how many other such patients exist, but this should be explored in subsequent studies. The final highly important finding from this study is that PCR performed directly on plasma in the absence of microbial isolation can be used to demonstrate superantigen genes.

## Data Availability

The raw data used in our study (PCR) and its corresponding figure are contained within the manuscript. The figure has not been enhanced or altered in any way except to provide labels for wells. The data for Table 1 are contained in the Table.

## Abbreviations

*S. aureus*: *Staphylococcus aureus*
*S. epidermidis*: *Staphylococcus epidermidis*
*S. saprophyticus*: *Staphylococcus saprophyticus*
TSS: Toxic shock syndrome
TSST-1: Toxic shock syndrome toxin-1
SPEs: Streptococcal pyrogenic exotoxins
*tstH*: toxic shock syndrome toxin-1 gene
PT: Prothrombin time
U/L: Units per liter
PCR: Polymerase chain reaction
TMP-SMZ: Trimethoprim-sulfamethoxazole
SARS-CoV-2: COVID-19 virus
Ig: Immunoglobulin
MHC: Major histocompatibility complex
MW: Molecular weight

## References

[1] Shands KN (1980). Toxic-shock syndrome in menstruating women: association with tampon use and *Staphylococcus aureus* and clinical features in 52 cases. N Engl J Med.

[2] Davis JP, Chesney PJ, Wand PJ, LaVenture M (1980). Toxic-shock syndrome: epidemiologic features, recurrence, risk factors, and prevention. N Engl J Med.

[3] Stevens DL (1989). Severe group a streptococcal infections associated with a toxic shock-like syndrome and scarlet fever toxin a. N Engl J Med.

[4] Cone LA, Woodard DR, Schlievert PM, Tomory GS (1987). Clinical and bacteriologic observations of a toxic shock-like syndrome due to *streptococcus pyogenes*. N Engl J Med.

[5] Belani K, Schlievert PM, Kaplan EL, Ferrieri P (1991). Association of exotoxin-producing group a streptococci and severe disease in children. Pediatr Infect Dis J.

[6] Wagner JG (1996). Acute group G streptococcal myositis associated with streptococcal toxic shock syndrome: case report and review. Clin Infect Dis.

[7] Spaulding AR (2013). Staphylococcal and streptococcal superantigen exotoxins. Clin Microbiol Rev.

[8] McCormick JK, Yarwood JM, Schlievert PM (2001). Toxic shock syndrome and bacterial superantigens: an update. Annu Rev Microbiol.

[9] Schlievert PM, Gocke JE, Deringer JR (1993). Group B streptococcal toxic shock-like syndrome: report of a case and purification of an associated pyrogenic toxin. Clin Infect Dis.

[10] Schlievert PM, Shands KN, Dan BB, Schmid GP, Nishimura RD (1981). Identification and characterization of an exotoxin from *Staphylococcus aureus* associated with toxic-shock syndrome. J Infect Dis.

[11] Schlievert PM (2019). Staphylococcal superantigens stimulate epithelial cells through CD40 to produce chemokines. MBio.

[12] Schlievert PM, Kim MH (1991). Reporting of toxic shock syndrome *Staphylococcus aureus* in 1982 to 1990. J Infect Dis.

[13] Schlievert PM, Bettin KM, Watson DW (1977). Purification and characterization of group a streptococcal pyrogenic exotoxin type C. Infect Immun.

[14] Kreiswirth BN, Schlievert PM, Novick RP (1987). Evaluation of coagulase-negative staphylococci for ability to produce toxic shock syndrome toxin 1. J Clin Microbiol.

[15] Stach CS, Vu BG, Schlievert PM (2015). Determining the presence of superantigens in coagulase negative staphylococci from humans. PLoS One.

[16] Madhusoodanan J (2011). An enterotoxin-bearing pathogenicity island in *Staphylococcus epidermidis*. J Bacteriol.

[17] Krause RM, Rammelkamp CH, Denny FW, Wannamaker LW (1962). Studies of the carrier state following infection with group a streptococci. 1. Effect of climate. J Clin Invest.

[18] Salgado-Pabón W, Case-Cook LC, Schlievert PM (2014). Molecular analysis of staphylococcal superantigens. Methods Mol Biol.

[19] Bohach GA, Hovde CJ, Handley JP, Schlievert PM (1988). Cross-neutralization of staphylococcal and streptococcal pyrogenic toxins by monoclonal and polyclonal antibodies. Infect Immun.

[20] Becker K, Heilmann C, Peters G (2014). Coagulase-negative staphylococci. Clin Microbiol Rev.

[21] Hovelius B, Mardh PA (1984). *Staphylococcus saprophyticus* as a common cause of urinary tract infections. Rev Infect Dis.

[22] Kallstrom G (2011). Recovery of a catalase-negative Staphylococcus epidermidis strain in blood and urine cultures from a patient with pyelonephritis. J Clin Microbiol.

[23] McDonald JA, Lohr JA (1994). *Staphylococcus epidermidis* pyelonephritis in a previously healthy child. Pediatr Infect Dis J.

[24] Kanai H, Sato H, Takei Y (2014). Community-acquired methicillin-resistant *Staphylococcus epidermidis* pyelonephritis in a child: a case report. J Med Case Rep.

[25] Hall DE, Snitzer JA (1994). *Staphylococcus epidermidis* as a cause of urinary tract infections in children. J Pediatr.

[26] Chesney PJ, Davis JP, Purdy WK, Wand PJ, Chesney RW (1981). Clinical manifestations of toxic shock syndrome. JAMA.

[27] Rosene KA, Copass MK, Kastner LS, Nolan CM, Eschenbach DA (1982). Persistent neuropsychological sequelae of toxic shock syndrome. Ann Intern Med.

[28] Harrison LM, Morris JA, Lauder RM, Telford DR (2009). Staphylococcal pyrogenic toxins in infant urine samples: a possible marker of transient bacteraemia. J Clin Pathol.

[29] Araki M, Kariyama R, Monden K, Tsugawa M, Kumon H (2002). Molecular epidemiological studies of *Staphylococcus aureus* in urinary tract infection. J Infect Chemother.

[30] Giantonio BJ (1997). Superantigen-based immunotherapy: a phase I trial of PNU-214565, a monoclonal antibody-staphylococcal enterotoxin a recombinant fusion protein, in advanced pancreatic and colorectal cancer. J Clin Oncol.

[31] Schlievert PM, Peterson ML (2012). Glycerol monolaurate antibacterial activity in broth and biofilm cultures. PLoS One.

[32] Fast DJ, Schlievert PM, Nelson RD (1988). Nonpurulent response to toxic shock syndrome toxin 1-producing *Staphylococcus aureus*. Relationship to toxin-stimulated production of tumor necrosis factor. J Immunol.

[33] Brosnahan AJ, Schlievert PM (2011). Gram-positive bacterial superantigen outside-in signaling causes toxic shock syndrome. FEBS J.

[34] Stach CS (2016). Novel tissue level effects of the *Staphylococcus aureus* enterotoxin gene cluster are essential for infective endocarditis. PLoS One.

